# Function and mechanism of TREM2 in bacterial infection

**DOI:** 10.1371/journal.ppat.1011895

**Published:** 2024-01-18

**Authors:** Zehua Wu, Shiyue Yang, Xiangming Fang, Qiang Shu, Qixing Chen

**Affiliations:** 1 Department of the Clinical Research Center, Children’s Hospital, National Clinical Research Center for Child Health, Zhejiang University School of Medicine, Hangzhou, People’s Republic of China; 2 Department of Anesthesiology, First Affiliated Hospital of Soochow University, Soochow, People’s Republic of China; 3 Department of Anesthesiology and Intensive Care, The First Affiliated Hospital, Zhejiang University School of Medicine, Hangzhou, People’s Republic of China; 4 Key Laboratory of Diagnosis and Treatment of Neonatal Diseases of Zhejiang Province, Hangzhou, People’s Republic of China; University of Queensland, AUSTRALIA

## Abstract

Triggering receptor expressed on myeloid cells 2 (TREM2), which is a lipid sensing and phagocytosis receptor, plays a key role in immunity and inflammation in response to pathogens. Here, we review the function and signaling of TREM2 in microbial binding, engulfment and removal, and describe TREM2-mediated inhibition of inflammation by negatively regulating the Toll-like receptor (TLR) response. We further illustrate the role of TREM2 in restoring organ homeostasis in sepsis and soluble TREM2 (sTREM2) as a diagnostic marker for sepsis-associated encephalopathy (SAE). Finally, we discuss the prospect of TREM2 as an interesting therapeutic target for sepsis.

## Introduction

Since the identification of triggering receptor expressed on myeloid cells 1 (TREM1) in 2000, the TREMs gene family has grown largely. In humans, *Trem1*, *Trem2*, TREM-like transcript 1 (*Treml1*) and *Treml2* were described. In mice, additional members such as *Trem3*, *Trem4*, *Trem5*, and *Treml6* were detected. The genes encoding TREMs are located on human chromosome 6p21.1 and mouse chromosome 17 and are highly evolutionarily conserved [1,2]. TREM2 is found broadly in myeloid-derived cells, including microglial cells [3], dendritic cells [4], osteoclasts [5], and resident macrophages in tissues such as adipose tissue, skin, gut, liver, and the pulmonary alveolus [6–10]. Increasing attention has focused on TREM2 following the discovery of Nasu–Hakola disease (NHD), which is characterized by early frontotemporal dementia-like syndrome with polycystic osseous lesions due to homozygous mutations in TREM2 such as Y38C, T66M, and V126G that impact its folding and stability [11–14]. Several independent genome-wide sequencing studies further showed that individuals with the H157Y mutation, the rare R47H mutation, and the R62H mutation in *Trem2* gene [15,16], which impair binding to phospholipid ligands [17,18], are susceptible to Alzheimer disease (AD). Other TREM2 mutants, such as N68K and D87N, have also been found in patients with AD, but they are so rare in the population and their risk remains uncertain [14]. In addition to central nervous system diseases, TREM2 also participates in inflammatory diseases, such as cirrhosis [19], inflammatory bowel disease [8], chronic obstructive pulmonary disease [20], multiple sclerosis [21], stroke [22], cholestasis [23], sepsis [24], acne [25], and leprosy granulomas [26].

TREM2 plays important roles in regulating cell survival, proliferation, migration, phagocytosis, energy metabolism and the secretion of cytokines and chemokines, and was implicated in immunosuppressive functions mediated by tumor-associated macrophages [1,27]. TREM2 also presents as a soluble form, sTREM2, which can not only serve as biomarker for diseases but also promote cell survival as well [3]. In this review, we particularly focus on the mechanisms behind phagocytosis and killing of bacteria, regulating inflammatory responses and metabolic homeostasis by TREM2. In addition, we discuss the therapeutic prospect of TREM2 in infectious diseases such as sepsis.

## Structure and downstream pathways of TREM2

The structure of TREM2 consists of a short stalk region that spans the cytomembrane and connects the cytoplasmic tail which has no downstream signaling activation function, to a V-type immunoglobulin domain which contains the functional region for ligand binding. As a cell surface receptor, TREM2 binds to a wide range of lipids, including phospholipids [14,28], myelin sulfatide [29], lipid components exposed during myelin injury [30], lipoprotein, apolipoprotein E (APOE), apolipoprotein J (APOJ), and low density lipoprotein (LDL) [31,32]. TREM2 also binds to lipid components on bacterial cell walls, such as lipopolysaccharide (LPS) on *Escherichia coli* [33], nonglycosylated mycolic acid on mycobacterium [34], and lipooligosaccharide on the outer membrane of *Neisseria gonorrhoeae* [35]. However, the structural basis for lipid binding remains unclear. Based on the available information, lipid binding may partly be achieved by electrostatic interactions. The hydrophobic and cationic regions in the extracellular segment of TREM2 can bind to the polar head and the acyl chains of phospholipids [14], and the hydrophobic and cationic regions in the extracellular segment of TREM2 are mainly composed of arginine. When arginine is mutated at residue 47 (R47H) or an electrolytic substance is added externally, the binding of TREM2 to the anionic ligand is reduced [32,36,37]. Exogenous addition of anionic bacterial carbohydrates, particularly dextran sulfate, lipopolysaccharides, and lipophosphocholic acid, can competitively inhibit the binding of TREM2 to bacteria [33].

When ligands bind to TREM2, the SRC family kinases phosphorylate tyrosine residues in the immunoreceptor tyrosine activator motif (ITAM) of DNAX activation protein 12 (DAP12) or DAP10, which bind electrostatically to the cell membrane region of TREM2, leading to the recruitment of splenic tyrosine kinase (SYK). The TREM2-DAP12/DAP10 signaling pathway triggers a phosphorylation cascade that leads to the activation or inhibition of a number of pathways that regulate cell functions [1,38,39]. TREM2 activates different signaling pathways in response to bacterial infection, which will be described in detail below.

## TREM2-mediated bacterial engulfment

As a phagocytic receptor, TREM2 facilitates the uptake of apoptotic neuronal cells, damaged myelin, and A-β plaques by microglia, which blocks the production of inflammatory cytokines in the central nervous system [18,21,31,40]. Initial studies revealed the involvement of TREM2 in the engulfment of bacteria through expression of the TREM2-DAP12 complex in nonphagocytic Chinese hamster ovary (CHO) cells, which promoted the binding and phagocytosis of bacteria [41]. TREM2 mediates the internalization of bacteria such as *E. coli*, *Francisella tularensis*, *Staphylococcus aureus*, *Pseudomonas aeruginosa*, *Mycobacterium tuberculosis*, and *Brucella abortus* [33,34,41]. The effects of TREM2 expression in different experimental models on phagocytosis and clearance of different bacteria, as well as on the produnction of inflammatory factors, were shown in Table 1.

**Table 1:** The role of TREM2 in regulating bacterial phagocytosis, clearance and the release of inflammatory factors^α^.

Mice/cell	Loss/gain of TREM2 function	Bacteria	Effect on bacterial phagocytosis	Effect on bacterial clearance	Effect on inflammatory factors	Reference
Mice	Deficiency	*M*. *tuberculosis*	-	enhanced	enhanced levels of TNF-α and MCP-1	[42]
Mice	Deficiency	*B*. *pseudomallei*	not affected	enhanced	reduced levels of TNF-α, IL-1β and IL-6	[43]
Mice	Deficiency	*E*. *coli*	-	-	enhanced levels of IL-6, KC and MCP-1	[44]
Mice	Deficiency	*S*. *pneumoniae*	-	enhanced	reduced levels of TNF-α, IL-1β, IL-6 and MCP-1	[45]
Mice	Deficiency	*P*. *aeruginosa*	-	reduced	enhanced levels of IL-1β and IL-18	[46]
Mice	Deficiency	*K*. *pneumoniae*	-	not affected	-	[47]
BMDM	Deficiency	*K*. *pneumoniae*	reduced	-	enhanced levels of TNF-α and KC	[47]
BMDM	Deficiency	*M*. *tuberculosis*	not affected	enhanced	-	[42]
BMDM	Deficiency	*S*. *pneumoniae*	-	-	enhanced levels of TNF-α and KC	[45]
BMDM	Deficiency	*P*. *aeruginosa*, *S*. *aureus*, *S*. *pneumonia*, *E*. *coli*	-	reduced	-	[48]
BMDM	shRNA silencing	*B*. *abortus*	reduced	enhanced	enhanced level of TNF-α	[49]
BMDM	shRNA silencing	*E*. *coli*	reduced	-	-	[41]
BMDM	Overexpressing	*E*. *coli*	enhanced	enhanced	reduced level of IL-1β	[50]
RAW264.7	siRNA silencing	*P*. *aeruginosa*	-	-	enhanced levels of TNF-α, IL-1β and MIP-2	[51]
RAW264.7	siRNA silencing	*P*. *aeruginosa*	not affected	reduced	-	[52]
RAW264.7	Overexpressing	*E*. *coli*	-	-	reduced level of IL-6	[44]
RAW264.7	Overexpression	*P*. *aeruginosa*	not affected	enhanced	-	[52]
Lamina propria dendritic cell	Deficiency	*S*. *Typhimurium*	not affected	reduced	reduced level of IL-1β	[8]
Peritoneal macrophage	Deficiency	*E*. *coli*	reduced	-	enhanced levels of TNF-α and IL-6	[44]
Alveolar macrophage	Deficiency	*S*. *pneumoniae*	enhanced	-	reduced levels of TNF-α and KC	[45]
THP-1 cell	Deficiency	*M*. *tuberculosis*	reduced	enhanced	enhanced levels of TNF-α and IL-1β	[34]
CHO cell	Overexpression	*E*. *coli*, *F*. *tularensis*, *S*. *aureus*, *P*. *aeruginosa*	enhanced	-	-	[41]
Human macrophage	Overexpression	*C*. *acnes*	enhanced	-	enhanced level of IL-18	[25]

^α^ TNF-α, tumor necrosis factor; MCP, monocyte chemotactic protein; IL, interleukin; KC, keratinocyte chemoattractant; BMDM, bone marrow-derived macrophages; CHO, Chinese hamster ovary; MIP, macrophage inflammatory protein.

**Fig 1:**
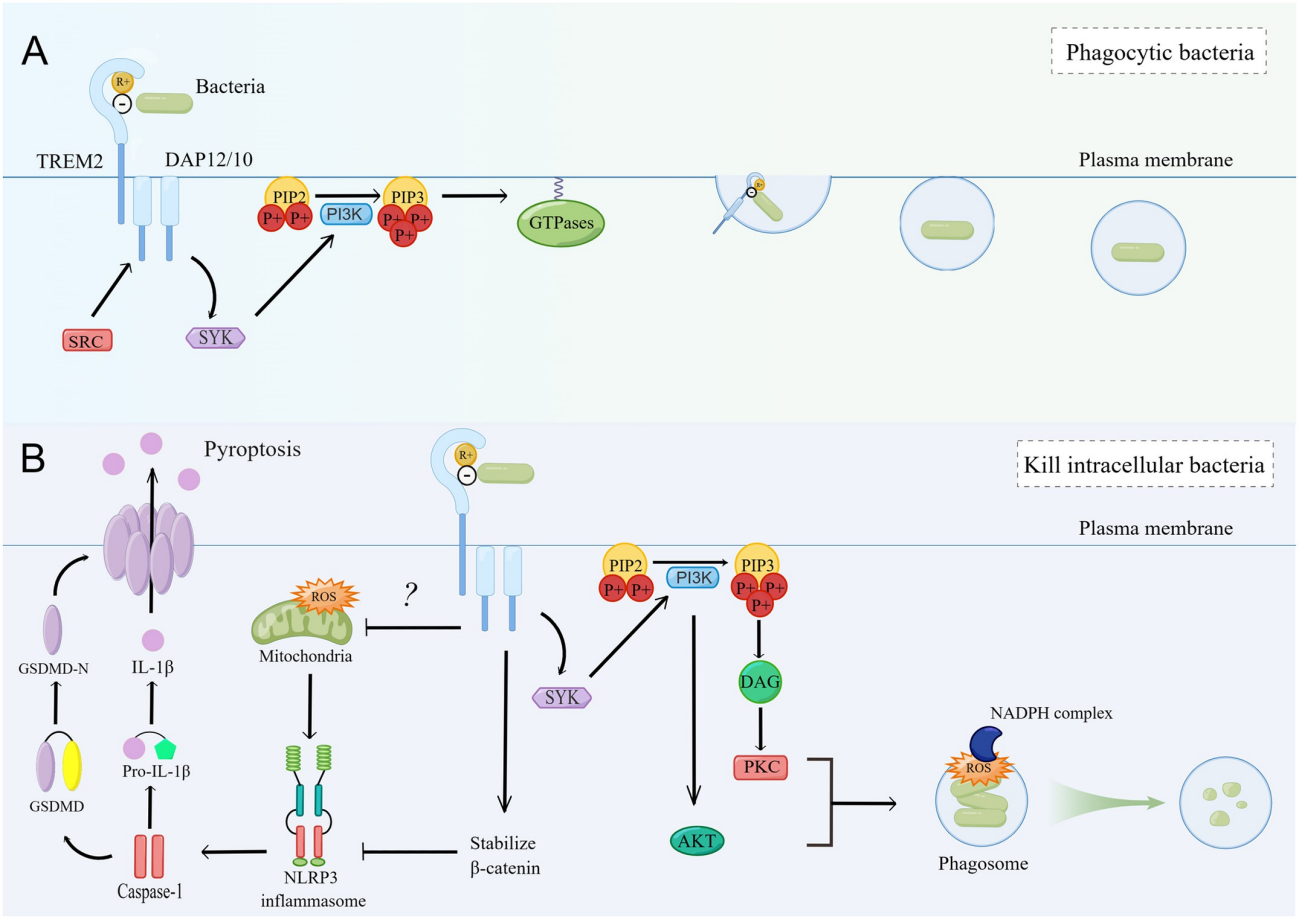
The role of TREM2 in bacterial infection. (A) TREM2 binds to lipid components on bacteria via the hydrophobic and cationic regions of extracellular fragments and progressively activates SYK, PI3K, and GTPases via the ITAM motif of DAP12. GTPases rearrange of actin-rich formation near the bacteria, allowing the bacteria to be internalized by the surrounding membrane [41,53]. (B) TREM2 inhibits NLRP3 inflammasome transcription and assembly by stabilizing β-catenin and reducing mitochondrial ROS release, which in turn inhibits cell pyroptosis and IL-1β release. In addition, AKT and PKC downstream of TREM2 can stimulate NADPH oxidase complex on phagosomes to release ROS, promoting bacterial killing [46,48,50]. The illustration rendering portion of this work was supported by Figdraw (https://www.figdraw.com/). TREM2, triggering receptor expressed on myeloid cells 2; SYK, splenic tyrosine kinase; PI3K, phosphoinositide 3-kinase; ITAM, immunoreceptor tyrosine activator motif; DAP12, DNAX activation protein 12; NLRP3, NACHT, LRR, and PYD domains-containing protein 3; ROS, reactive oxygen species; IL, interleukin; NADPH, nicotinamide adenine dinucleotide phosphate; GSMDM, gesdermin D; PIP2, phosphatidylinositol-4,5-bisphosphate; PIP3, phosphatidylinositol-3,4,5-trisphosphate; DAG, diacylglycerol; AKT, protein kinase B; PKC, protein kinase C.

TREM2 is a receptor on the cell membrane that activates a downstream signaling pathway through the ITAM motif of DAP12/DAP10. The signaling pathway of TREM2 involved in bacterial phagocytosis is shown in Fig 1A. ITAM mutations prevent its phosphorylation, allowing TREM2 to continue binding to bacteria but failing to mediate phagocytosis. Furthermore, inhibiting signaling pathways or factors downstream of TREM2-DAP12-ITAM, such as SRC family kinases, phosphoinositide 3-kinase (PI3K) or SYK, and actin polymerization, weakened the phagocytosis of bacteria by cells [41,53]. The use of an inhibitor of the Rho family of GTPases was also shown to reduce the uptake of *E*. *coli* by TREM2-DAP12 CHO cells, suggesting that PI3K-induced Rho family GTPases may play an important role in TREM2-mediated bacterial phagocytosis. GTPases are involved in the extension of pseudopods during phagocytosis, promoting the formation of actin-rich rearrangements that allow the particles to be internalized by the surrounding membrane [54]. In addition, Rac and Cdc42 may also be involved in pseudopod extension and membrane expansion of TREM2 during bacterial phagocytosis [41,53].

Of note, the absence of TREM2 does not affect the ability of immune cells to phagocytize bacteria in some cases, such as that murine bone marrow-derived macrophages (BMDMs) with low level of TREM2 can still effectively phagocytize mycobacteria [42]. The phagocytosis process in phagocytes is mediated by multiple receptors, and the effect of the loss of 1 receptor (TREM2) on phagocytosis may be compensated by the other receptors and the cascade of activation signals that these receptors lead to. DAP12, which works with TREM2 to initiate downstream signaling, also works in tandem with other receptors, such as TREM1 and myeloid DAP12-associating lectin-1 [55]. Whether the loss of TREM2 affects the cooperation of DAP12 with other receptors is unclear. In addition, the downstream molecular mechanism by which TREM2 regulates immune cells to phagocytic bacteria and other molecules still needs to be further explored.

## TREM2-mediated bacterial removal

TREM2 mediated intracellular signaling is crucial for bacterial clearance. TREM2 is expressed in phagocytes and takes part in removing invasive pathogens by regulating reactive oxygen species (ROS) production and pyroptosis (Fig 1B). In response to bacterial invasion, macrophages generate ROS via phagosomal nicotinamide adenine dinucleotide phosphate (NADPH) oxidase to protect against microbial pathogens [56,57]. The PI3K-protein kinase B (PKB/AKT) and protein kinase C (PKC) pathways downstream of TREM2-DAP12/DAP10 play a critical role in ROS generation [53,58,59]. In the early stage of bacterial challenge, macrophages with high level of TREM2 produce increased phagosomal NADPH oxidase 2 (NOX2)-derived ROS, which accelerate the killing of *Salmonella Typhimurium*, *E. coli*, and *P. aeruginosa* [50,52,53]. Furthermore, TREM2 suppresses the generation of mitochondria-derived ROS, which further inhibits the NACHT, LRR, and PYD domains-containing protein 3 (NLRP3)/caspase-1 activation to protect macrophages from pyroptotic death [48,50]. In response to *E*. *coli* challenge, BMDMs with low level of TREM2 show severely disrupted mitochondrial morphology, which may be related to the production of mitochondrial ROS [50]. TREM2 can inhibit pyogenic bacteria-induced macrophage pyroptosis to promote bacterial eradication [46,48]. In response to *P*. *aeruginosa* invasion, TREM2 is expressed on corneal resident macrophages and improves corneal pathology by reducing macrophage death and reducing the release of the inflammatory mediator interleukin-1β (IL-1β). Interestingly, coimmunoprecipitation of NLRP3 with TREM2 showed that TREM2 could mediate inflammasome activation through direct interactions [46]. A recent bacterial removal study based on cell experiments showed that TREM2 inhibited NLRP3 transcription in the nucleus and inflammasome complex assembly in the cytoplasm by stabilizing β-catenin. Knockout of TREM2 attenuated the activation of β-catenin [48].

**Fig 2:**
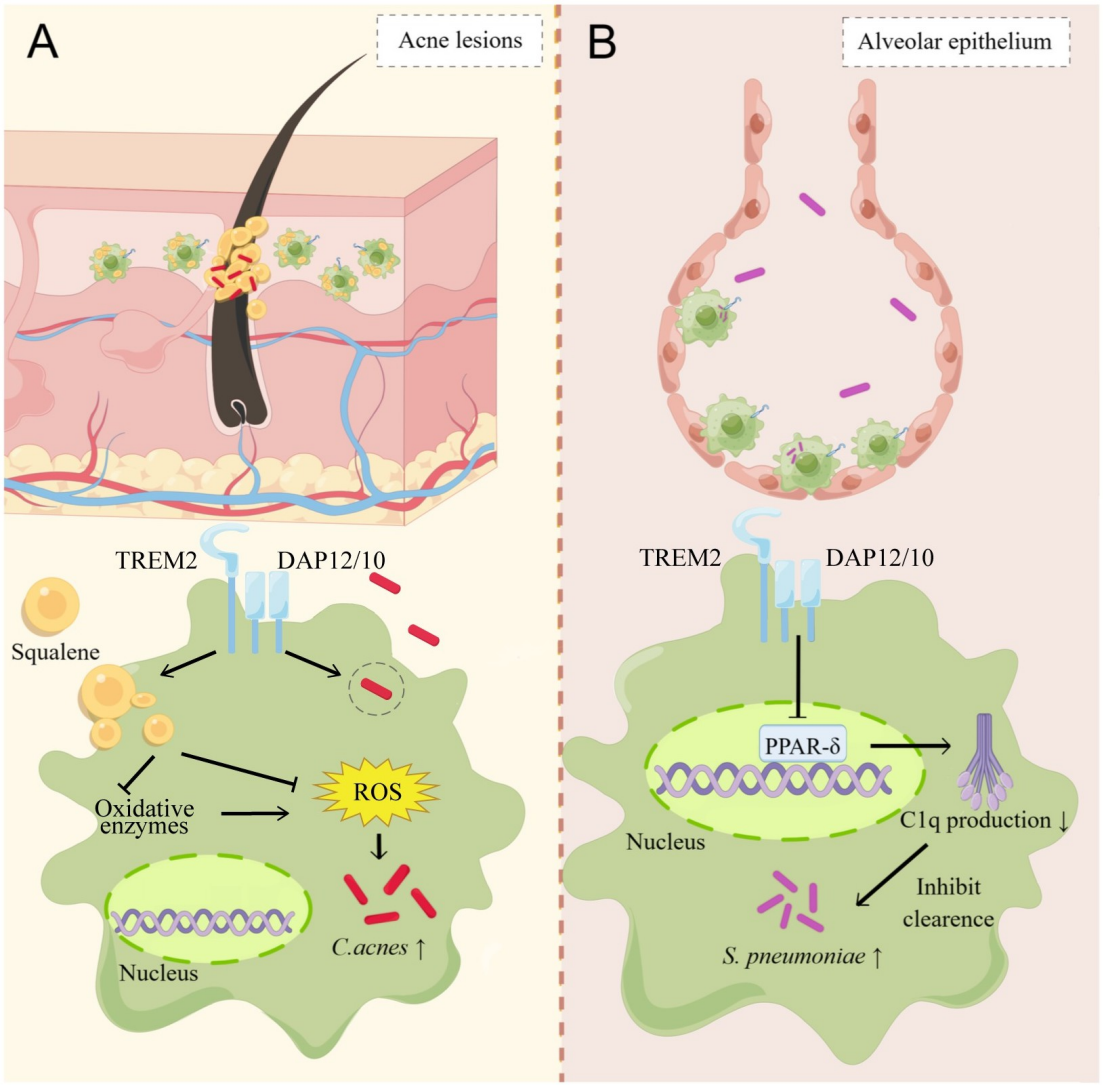
The antimicrobial effects of TREM2 on barrier immune cells. (A) TREM2 expression in acne lesions enhances the phagocytic capacity of macrophages against lipids and bacteria, but the macrophages do not facilitate microbial bacterial clearance due to the ability of squalene to clear ROS and to inhibit ROS production [25]. (B) TREM2 inhibits C1q transcription and basal C1q production by suppressing PPAR-δ activity in AMs, and C1q was up-regulated in *Trem2*^-/-^ AMs to enhance macrophage phagocytosis [45]. The illustration rendering portion of this work was supported by Figdraw (https://www.figdraw.com/). TREM2, triggering receptor expressed on myeloid cells 2; ROS, reactive oxygen species; C1q, complement 1q; PPAR-δ, peroxisome proliferator-activated receptor-δ; AMs, alveolar macrophages; DAP12, DNAX activation protein 12.

However, TREM2 may have different effects on immune cells in specific sites and pathogens, which may be due to different mechanisms. In a model of acne lesions caused by *Cutibacterium acnes*, excess production of squalene by hair follicle epithelium increases TREM2 expression in surrounding macrophages and induces TREM2-positive macrophages to enhance the phagocytosis of lipids and *C*. *acnes*. In this case, the macrophages with up-regulated level of TREM2 did not reduce the bacterial load. This is because that squalene in acne lesions is an ROS scavenger that removes ROS from macrophages, thus blocking the antibacterial response of macrophages (Fig 2A) [25]. TREM2 expression in alveolar macrophages (AMs) has a deleterious effect on pneumococcal pneumonia. *Trem2*^-/-^ mice showed enhanced lung bacterial clearance, reduced bacteremia, and improved survival. *Trem2*-deficient AMs showed increased phagocytosis in vivo and in vitro [45]. TREM2 has also been shown to be detrimental in the pulmonary infection model caused by *Burkholderia pseudomallei*, and the bacterial load in *Trem2*^-/-^ mice was significantly reduced relative to that in wild-type (WT) mice [43]. TREM2 may inhibit complement 1q (C1q) transcription and basal C1q production by suppressing peroxisome proliferator-activated receptor-δ (PPAR-δ) activity in AMs, and whole-gene transcriptome analysis showed that C1q was up-regulated in *Trem2*^-/-^ AMs to enhance macrophage phagocytosis (Fig 2B). The phagocytic difference between *Trem2*^-/-^ AMs and WT AMs was eliminated after the administration of C1q antibody, and exogenous C1q supplementation increased the phagocytic ability of WT AMs [45]. But there is no evidence that TREM2 can interact directly with PPAR-δ and subsequent studies showed no difference in C1q levels in *Trem2*^-/-^ and WT peritoneal-derived macrophages, further suggesting that TREM2 may have different regulatory mechanisms in resident macrophages in different tissues [44].

**Fig 3:**
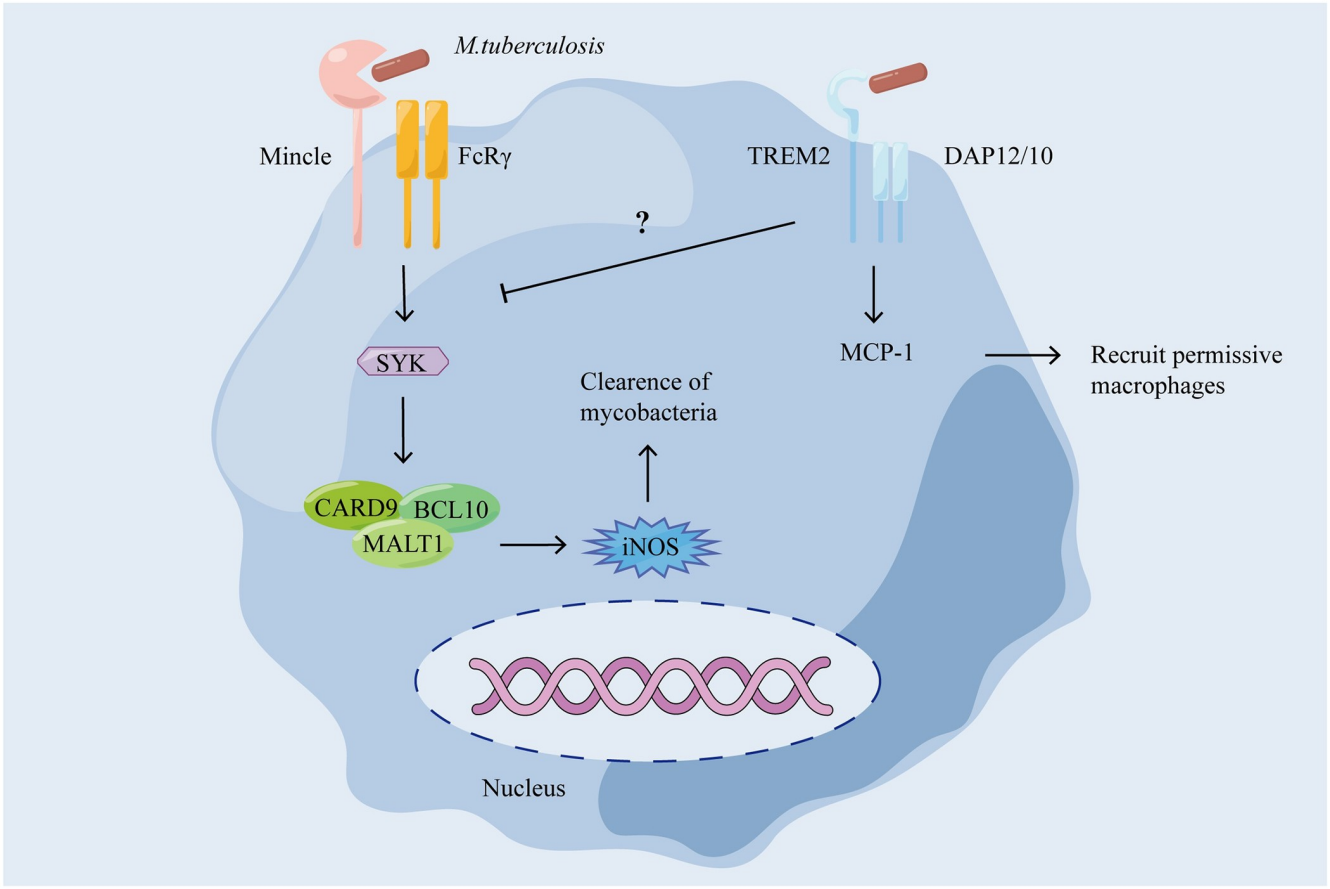
Mechanism of immune escape by mycobacteria via TREM2 in mice. TREM2 inhibits the Mincle-FcRγ-CARD9 pathway and reduces iNOS to kill bacteria. On the other hand, TREM2 promotes the synthesis and release of monocyte chemotactic protein (MCP)-1 to recruit permissive macrophages to enhance infectivity [42]. The illustration rendering portion of this work was supported by Figdraw (https://www.figdraw.com/). TREM2, triggering receptor expressed on myeloid cells 2; CARD9, caspase-recruitment domain family member 9; SYK, splenic tyrosine kinase; DAP12, DNAX activation protein 12; BCL10, B cell lymphoma 10; iNOS, inducible nitric oxide synthase; MALT1, mucosa-associated lymphoid tissue translocation protein 1.

In addition, some bacteria utilize TREM2 to invade immune cells and evade phagocyte antimicrobial immunity. *B*. *abortus* uses TREM2 receptor facilitating its entry into cells and inhibiting nitric oxygen production in macrophages to improve its intracellular survival [49]. *M*. *tuberculosis* utilizes TREM2 to inhibit the production of inducible nitric oxide synthase (iNOS) through restrict Mincle-FcRγ-caspase-recruitment domain family member 9 (CARD9)-mediated antimycobacterial immunity for immune escape in mice (Fig 3) [42,55]. Alternatively, live *M*. *tuberculosis* can up-regulate of TREM2 in a human macrophage-like cell-line, THP-1. Then, increased TREM2 signaling induces a type I interferon (IFN) response responsible for SYK-independent ROS generation, leading to increased intracellular survival of mycobacteria [34]. Current studies had shown that TREM2-mediated bacterial clearance is closely related to the production of intracellular ROS and nitric oxide. However, the role of TREM2 in regulating ROS production in different bacterial diseases is contradictory. Intracellular ROS production is dependent on TREM2 and is responsible for the removal of *S*. *typhimurium* or *P*. *aeruginosa* [52,53], while in infection with *M*. *tuberculosis* and *E*. *coli*, overexpression of TREM2 leads to a decrease in ROS [34,50]. The reason for this phenomenon may be that TREM2 has the function of promoting and inhibiting the production of ROS at the same time, and different bacteria may use TREM2 to drive different reactions, such as *M*. *tuberculosis* uses TREM2 to reduce intracellular ROS and promote its survival in the cell. Importantly, the source of intracellular ROS production mediated by TREM2 is not clear, and identifying the source of intracellular ROS after bacterial infection is helpful to study the regulatory role of TREM2 on ROS. In addition, multiple gene analysis datasets reveal that genes regulated by TREM2 are significantly correlated with phagosome and lysosome functions [25,29]. It is also worth investigating whether lysosomes regulated by TREM2 during infection play a role in the TREM2 antimicrobial response in the future.

## TREM2 orchestrates the TLR response

Preliminary findings on sepsis showed that TREM1 amplifies systemic inflammatory response syndrome [60]; in contrast, TREM2 negatively regulates TLRs. TLRs are activated by pathogen-associated molecular patterns (PAMPs) and damage-associated molecular patterns (DAMPs), leading to the release of inflammatory mediators [61]. Especially in the case of severe infection, TLRs are overactivated, and the host develops a syndrome characterized by an inflammatory cytokine storm and multiple tissue and organ dysfunction, which is known as sepsis [62]. TREM2 can inhibit the release of inflammatory cytokines by immune cells. BMDMs, thioglycolate-recruited peritoneal macrophages, and dendritic cells from *Trem2* knockout mice produce more inflammatory cytokines than WT macrophages in response to the TLR ligands LPS, cytosine-phosphate-guanine (CpG), and zymosan [44,63,64]. The mechanism by which TREM2 inhibits LPS-mediated inflammation in vivo is complex and has not been thoroughly studied. Inhibiting inflammatory cytokine release by TREM2-DAP12 may require activation of the downstream SYK pathway [65]. In vitro studies showed that inhibition of the LPS response by DAP12 may be mediated by the adaptor protein docking protein 3 (DOK3). In response to LPS stimulation, the ITAM motif of DAP12 binds directly to the phosphotyrosine-binding domain on DOK3, and then DOK3 is phosphorylated and localized on the membrane. Phosphorylated DOK3 forms a complex with growth factor receptor-bound protein 2—son of sevenless 1 to inhibit extracellular signal-regulated kinase (ERK) phosphorylation and subsequent inflammatory cytokine production [65,66]. However, peritoneal macrophages stimulated in vivo by LPS did not show a correlation between DOK3 and DAP12, and the study used low-dose LPS stimulation (≤1 ng/ml). During gram-negative bacterial infection, the LPS concentration could be much higher than this.

In contrast, TREM2 expression on specific cell types promotes the release of proinflammatory cytokines. Proinflammatory cytokines were significantly decreased in the lung homogenate and bronchoalveolar lavage fluid of *Trem2*-deficient mice [43]. In a mouse model of inflammatory bowel disease, TREM2 expression on intestinal lamina propria-dendritic cells promoted downstream TLR signaling. In response to stimulation by LPS and CpG, *Trem2*-deficient lamina propria-dendritic cells secreted less IL-1, IL-6, and IL-12p70 than lamina propria-dendritic cells isolated from WT mice [8]. In a skin acne model, TREM2 expression in macrophages contributes to inflammation by up-regulating the expression of proinflammatory chemokines to recruit and activate immune cells [25]. Worth mentioned is that these 3 kinds of cells are located in the lung, intestine, and skin, which are barrier cells in the host and have long been in contact with pathogenic microorganisms. One possible explanation is that macrophages in the lung need to regularly face and clear airborne pathogens, and appropriately elevated inflammation levels may facilitate AM clearance of invasive pathogens. Therefore, TREM2 expression on barrier immune cells exhibits unknown intracellular regulation to adapt to the pathogen-rich environment. During bacterial infection, TREM2 has a tendency to promote macrophage transformation to M2 type and alters macrophage cytokine production. In *P*. *aeruginosa*-induced keratitis, low expression level of TREM2 leads to down-regulation of Th2 cytokines IL-4, IL-5, and IL-10, in contrast to up-regulation of Th1 cytokines IL-12, IL-18, and IFN-γ [51]. The role of TREM2 in promoting the anti-inflammatory phenotype of macrophages during bacterial infection is unclear. In inflammation-related diseases, TREM2 protects tissue from inflammatory damage by mediating the anti-inflammatory phenotype of immune cells and promotes tissue repair. In stroke and in the LPS-induced encephalopathy, overexpression of TREM2 promoted M2-polarization in LPS-treated microglia, which alleviated neuroinflammation and reduced the number of apoptotic neuron [67,68]. In chronic obstructive pulmonary disease and colonic epithelial injury, TREM2 inhibits the expression of M1-activated markers and promotes M2 activation, which is important for colonic epithelial healing [20,69]. Overall, these studies support the possibility that TREM2 regulates the anti-inflammatory phenotype of immune cells during infection and mitigates inflammatory tissue damage.

## TREM2 affects neutrophil infiltration and antigen presenting function of immune cells

The production of pro-inflammatory cytokines and chemokines by pattern recognition receptors (such as TLRs) after pathogen recognition plays an important role in initiating early innate immune responses and host defense. During infection, TREM2 inhibits TLR responses and reduces pro-inflammatory cytokines and chemokines, as well as recruitment of neutrophils. In *E*. *coli*-induced pneumonia and peritonitis models, *Trem2*^-/-^ mice showed elevated levels of keratinocyte chemoattractant, a chemokine required for neutrophilic influx, and increased numbers of neutrophils [44,45]. Consistent with this, a large number of neutrophils were present at the site of infection in *Trem2*^-/-^ mice stimulated by *P*. *aeruginosa* and components of *M*. *tuberculosis* [42,51]. However, in the bacterial pneumonia model induced by *B. pseudomallei* and *Klebsiae pneumoniae* [43,47], the number of neutrophils did not increase in *Trem2*^-/-^ mice in the early stage of infection, suggesting that the effect of TREM2 on neutrophils recruitment is also bacteria specific. In addition, TREM2 expressed on antigen-presenting cells may affect the antigen-presenting function of immune cells. In vitro TREM2-deficient intestinal lamina propria-derived dendritic cells and cultured bone marine-derived dendritic cell showed reduced clearance of intracellular *S*. *typhimurium*, further attenuates the ability of *Trem2*^-/-^ dentritic cell to process relevant bacterial antigens and induce the proliferation of bacteria-associated antigen-specific T-cell [8]. The poor antigen presenting function may affect the subsequent clearance of bacteria. Taken together, these studies suggest that TREM2 not only affects phagocyte function, but also affects subsequent neutrophil recruitment and adaptive immune initiation. It has been reported that TREM2 may also expressed on neutrophils [70]. Whether the loss of TREM2 affects the function of recruited neutrophils and whether the neutrophils recruited to the site of infection have effect on the prognosis remains to be further studied.

## TREM2 maintains organ metabolic homeostasis in sepsis

TREM2 expressed on tissue-resident macrophages not only mounts an important defense against bacteria but also regulates metabolic coordination in parenchymal cells. Macrophages with impaired TREM2 expression in the liver release exosomes containing miR-106b-5p, thereby impairing mitochondrial structure and energy supply in hepatocytes. Increasing TREM2 expression in liver macrophages may improve the prognosis of mice with severe infection by improving the liver energy supply [71]. Excessive activation of inflammation leads to multiple organ failure. Patients with sepsis exhibit abnormal heart function (reduced ejection fraction), which is often referred to as sepsis-induced cardiomyopathy [72]. A recent study reported that TREM2 improved survival and restored cardiac function in septic mice. Mac1 cells, which are resident macrophages that express TREM2 between cardiomyocytes, is responsible for restoring cardiac homeostasis via the phagocytosis and removal of dysfunctional junk mitochondria expelled by cardiomyocytes. The loss of TREM2 reduces the self-maintenance ability of heart-resident Mac1 cells, leading to the accumulation of damaged mitochondria in the myocardium, which leads to injury to the heart and a decline in cardiac systolic function [24]. Of note, the current studies on regulation of parenchymal cell metabolism by TREM2 during infection mostly focused on TREM2-mediated phagocytosis to reduce cell production of harmful substances. Whether TREM2 may regulate metabolism by other means deserves further investigation.

## sTREM2 can serve as a biomarker in sepsis-associated encephalopathy

An interesting phenomenon is that TREM2 expression declines rapidly on the surface of myeloid cell membranes when stimulated by bacteria, IFN-γ or LPS, which is caused by the cleavage of TREM2 in the stalk area by a disintegrin and metalloproteinase domain-containing protein 17 (ADAM17) or ADAM10 [3,44,51,58]. A recent study showed that tumor necrosis factor-α (TNF-α) and IL-1β could induce proteolytic cleavage of TREM2 by activating ADAM17 and release of sTREM2. Preadministration of the ADAM17-specific inhibitor (TAPI) significantly counteracted the reduction in full-length TREM2 protein induced by TNF-α and IL-1β [73], which may explain the early decline in TREM2 protein expression in the early stage of myeloid cell contact with bacteria. In addition, there is evidence that TREM2 has a very short half-life and must be continually resynthesized to maintain baseline expression. The stimulation of LPS can cause the TREM2 transcription level on the myeloid cells declining, which leads to a rapid decline in TREM2 expression. Consistent with this phenomenon, the use of the translation inhibitor cyclohexylamine resulted in a rapid decline in TREM2 expression [44]. However, these studies were based on in vitro experiments, and the in vivo environment is much more complex and variable, making the early decline in TREM2 expression difficult to explain. After being released into the extracellular milieu, sTREM2 can be detected in cerebrospinal fluid and serum [74,75]. sTREM2 can be used as a biomarker for a variety of diseases and is elevated in the cerebrospinal fluid in patients with amyotrophic lateral sclerosis, nonalcoholic steatohepatitis, AD, and multiple sclerosis [76–80]. Central nervous system exposure to hyperinflammation during sepsis causes cognitive dysfunction and dementia, which is called sepsis-associated encephalopathy [81]. Substantially increased levels of sTREM2 were also found in the cerebrospinal fluid of SAE patients, and sTREM2 is considered to be a prognostic biomarker for cognitive decline in SAE patients [82].

However, further work will be necessary to address these questions so as to define the role of TREM2 on microglia in SAE and explore the bioactivity of sTREM2. It has been reported that elevated sTREM2 may be associated with changes in microglia activity in the brain [83] and sTREM2 can activate intracellular ERK1/2 and PI3K-AKT signaling pathway to protect immune cells form death during viral infection [20]. It is predictable that sTREM2 may have the same corresponding role in bacterial infection.

## TREM2 can be a new treatment target for sepsis

Given that TREM2 is emerging as a key signaling factor in numerous diseases, researchers are now focusing on targeting TREM2 for disease treatment. Injecting TREM2-overexpressing BMDMs into mice with pneumonia or peritonitis induced by *E*. *coli* can reduce the mortality and bacterial burden effectively in mice [50]. Similarly, in mice with cecal ligation and perforation-induced sepsis, early administration of TREM2-overexpressing BMDMs also improved survival, reduced bacterial load, and alleviated organ damage [84]. Moreover, TREM2 transgenic mice showed milder sepsis-induced organ dysfunction and less bacterial load [71]. More recently, antibodies that target TREM2 were developed and evaluated in the first phase II clinical study in AD. These antibodies activate downstream signaling pathways of TREM2 or increase membrane distribution of the receptor by reducing hydrolytic shedding of TREM2 proteins [85–88]. With the increasing number of clinical drug-resistant strain, targeting host immune cells to enhance their antibacterial ability becomes particularly important in sepsis. Thus, TREM2 should be a promising target for sepsis treatment in the near future.

## Conclusions

Much has been achieved in understanding the clearance function of TREM2 on a variety of pathogens since the discovery that TREM2 mediates immune cells to phagocytose bacteria, but many questions remain to be addressed. First, most of the infection-related findings have been performed in *Trem2* knockout mice, but TREM molecules are different between mice and humans. Though some clinical data suggest that TREM2 levels may be associated with sepsis severity in patients, clinical data from different populations and different countries are still needed to verify this association [50,84]. Currently, research has focused on the function of TREM2 at the innate immune level, such as the ability of TREM2 expressed on macrophages to engulf and eliminate bacteria. However, the role of TREM2 in other aspects such as that the effects of TREM2 on antigen presentation and complement system are poorly understood, which are needed to be futher explored. Evidence from protein interaction studies suggesting that TREM2 suppresses the classical complement cascades by binding to C1q [89]. Interestingly, C1q levels have previously been found to be reduced in TREM2-expressing alveolar macrophages [45]. It is unclear whether TREM2 reduces C1q in macrophages because TREM2 binds to C1q extracellular to reduce C1q entry into macrophages, or if TREM2 directly inhibits C1q transcription within cells. The complement system also play an important role in killing invading pathogens and further studies are needed to investigate the role of TREM2 in classical complement cascades in infectious models. Although sTREM2 levels are correlated with cognitive dysfunction in patients with SAE [82], the mechanism by which sTREM2 is released during sepsis and how it affects brain function remains to be understood. Moreover, there are still conflicting evidences on the role of TREM2 in pathogen removal. TREM2 promotes reducing bacterial burden by exerting host antibacterial responses during *S*. *typhimurium* and *P*. *aeruginosa* infection [52,53], while TREM2 facilitates the growth of *B*. *abortus* and *M*. *tuberculosis* within macrophages by inhibiting antibacterial ROS production [34,49]. The duality of TREM2 in regulating inflammation and killing bacteria in different tissues suggests a more complex role for TREM2 in infection diseases, future studies examining the hub role of the membrane bound TREM2 in different pathogens and the bioactivity of soluble forms of TREM2 in infectious diseases may yield significant insight into the role of TREM2 as a prognostic and diagnostic tool and its potential as therapeutic target.
